# Rheology of a Dilute Suspension of Aggregates in Shear-Thinning Fluids

**DOI:** 10.3390/mi11040443

**Published:** 2020-04-22

**Authors:** Marco Trofa, Gaetano D’Avino

**Affiliations:** Dipartimento di Ingegneria Chimica, dei Materiali e della Produzione Industriale, Università di Napoli Federico II, Piazza Giorgio Ascarelli 80, 80125 Napoli, Italy; marco.trofa@unina.it

**Keywords:** rheology, shear-thinning fluids, intrinsic viscosity, fractal aggregates, numerical simulations

## Abstract

The prediction of the viscosity of suspensions is of fundamental importance in several fields. Most of the available studies have been focused on particles with simple shapes, for example, spheres or spheroids. In this work, we study the viscosity of a dilute suspension of fractal-shape aggregates suspended in a shear-thinning fluid by direct numerical simulations. The suspending fluid is modeled by the power-law constitutive equation. For each morphology, a map of particle angular velocities is obtained by solving the governing equations for several particle orientations. The map is used to integrate the kinematic equation for the orientation vectors and reconstruct the aggregate orientational dynamics. The intrinsic viscosity is computed by a homogenization procedure along the particle orbits. In agreement with previous results on Newtonian suspensions, the intrinsic viscosity, averaged over different initial orientations and aggregate morphologies characterized by the same fractal parameters, decreases by increasing the fractal dimension, that is, from rod-like to spherical-like aggregates. Shear-thinning further reduces the intrinsic viscosity showing a linear dependence with the flow index in the investigated range. The intrinsic viscosity can be properly scaled with respect to the number of primary particles and the flow index to obtain a single curve as a function of the fractal dimension.

## 1. Introduction

Suspensions of solid particles are encountered in a variety of industrial and biological systems. The knowledge of the viscosity and, more in general, of the rheological properties of these materials is fundamental for correctly designing the processing stage and predicting the hydrodynamic resistance. It is well-known that the addition of solid particles in a fluid increases the viscosity as compared with the suspending liquid [1]. Particle shape and size, as well as the solid concentration strongly alter the suspension viscosity giving rise to non-Newtonian phenomena such as shear-thinning and normal stresses [1,2,3].

For the simplest case of a dilute suspension of spherical particles in a Newtonian fluid, Einstein calculated that the suspension viscosity is related to the fluid viscosity $\eta_{s}$ according to the formula $\eta_{s}\left(1+B\phi \right)$ where ϕ is the solid volume fraction and the factor *B*, usually referred as ‘intrinsic viscosity’, is equal to 2.5 [4,5]. Einstein’s calculation was extended by Jeffery to spheroidal particles [6], finding that the intrinsic viscosity depends on the particle orientation and aspect ratio. Specifically, the minimum and maximum average viscosities are obtained for particles aligned along the vorticity axis or tumbling on the flow-gradient plane, respectively. For randomly-oriented particles, integration of the instantaneous intrinsic viscosity for several initial orientations over the corresponding orbits leads to an average value of *B* higher than the Einstein’s coefficient for both prolate and oblate spheroids [7].

In many processes, however, the suspended particles have a complex and irregular shape, without symmetry axes or planes. This is, for instance, the case when primary spherical particles undergo an aggregation process and form clusters with fractal-like morphology [8,9,10]. As for the spheroidal particle case, the prediction of the intrinsic viscosity requires the calculation of the orientational dynamics of the particles subjected to an external shear flow. This approach is carried out in the work by Harshe and Lattuada [11] where the average rigid body resistance matrix of arbitrary shaped clusters made of uniform sized spheres is computed through the Stokesian dynamics method and Brownian dynamic simulations. The intrinsic viscosity was found to be similar to the spherical particle case for clusters with high fractal dimension, indicating no preferential orientation in the flow. Deviations from the Einstein’s coefficient was, instead, found for aggregates with low fractal dimension due to their more anisotropic shape.

All the aforementioned works deal with Newtonian suspensions. In several applications, however, the suspending fluid shows non-Newtonian properties such as shear-thinning and viscoelasticity. A typical example is in the tire industry where, during the processing stage, particles of carbon black or silica are added to a polymer melt and can agglomerate and form complex structures. It is well-known that fluid non-Newtonian properties alter the suspension rheology as compared to the Newtonian case [12,13]. For instance, for a dilute suspension of spherical particles in a power-law fluid, it has been shown that shear-thinning reduces the intrinsic viscosity [14,15]. Concerning more complex particle shapes, the rheology of a dilute suspension of spheroids in a generalized Newtonian fluid is recently investigated by numerical simulations [16]. Different flows of a Carreau fluid around spheroidal particles are simulated and a homogenization procedure is adopted to obtain the intrinsic viscosity of the suspension as function of the applied rate of deformation, thinning exponent and particle aspect ratio. The results show that the intrinsic viscosity strongly depends on the particle aspect ratio along with the rheological parameters of the constitutive equation [16]. Very recently, these calculations have been extended to a dilute suspension of rigid rods in a power-law fluid showing no similarity of the rheological coefficients between rods and spheroids with large aspect ratio [17]. Similar studies for dilute suspensions of particles with irregular shape, such as fractal-like morphologies, are not available.

In this paper, we investigate the rheology of a dilute suspension of aggregates with complex shape suspended in a shear-thinning fluid by direct numerical simulations. Aggregates made of primary spherical particles are built through a fractal-like model. The fluid is modeled by the power-law constitutive equation. The dynamics of a single particle in an unbounded shear flow field is first computed. To this aim, finite element simulations are employed to calculate the angular velocity of the particle for several orientations on the unity sphere. The orientational dynamics is then reconstructed by integrating the kinematic equations for the orientation vector interpolating the angular velocity field. The first-order contribution to the viscosity is computed by means of a homogenization procedure and time-averaged over the particle orbits. The effect of particle morphology and power-law index on the ensemble-average intrinsic viscosity is investigated.

## 2. Mathematical Model and Numerical Method

### 2.1. Governing Equations

We consider a dilute suspension of rigid, non-Brownian aggregates in a continuous shear flow. The computational domain consists in a single aggregate placed at the center of a spherical domain with radius much larger than the maximum size of the aggregate. A Cartesian reference frame is selected with *x*, *y*, and *z* denoting the flow, gradient, and vorticity directions, respectively. Shear flow boundary conditions are applied on the spherical surface whereas a rigid-body motion is imposed on the particle boundary. For the investigated problem (unbounded shear flow) the only relevant particle kinematic quantity is the angular velocity denoted by $\omega_{p}=d\theta_{p}/dt$ with $\theta_{p}$ the rotation angle.

**Figure 1:**
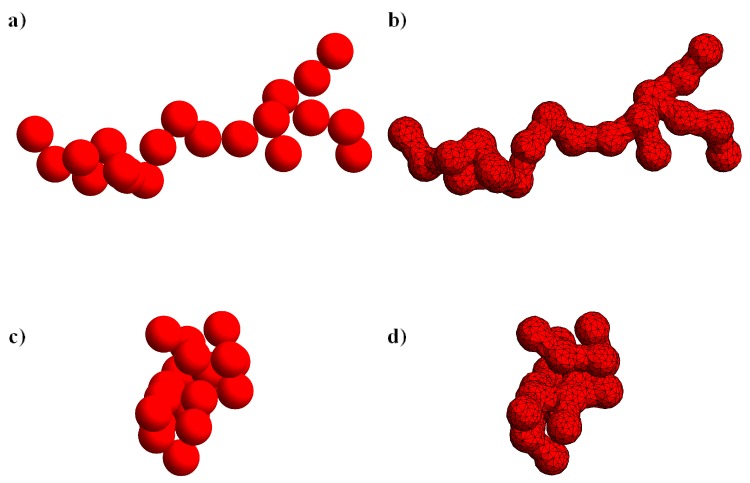
Examples of aggregate shapes obtained from the particle-cluster method for $N_{p}=20$, $k_{f}=1.3$, and $D_{f}=1.5$ (**a**) or $D_{f}=2.5$ (**c**). To avoid numerical issues in the region between tangent particles, the centers of the spheres in contact are connected with a set of cylinders with radius 0.732a. In panels (**b**) and (**d**) the final geometry of the aggregates and the surface mesh are shown.

The aggregate is made by primary spherical particles with radius *a*. The morphology is described by the following fractal equation [18]:

(1) $$N_{p}=k_{f}{\left(\frac{R_{g}}{a}\right)}^{D_{f}},$$

where $N_{p}$ is the number of primary particles, $R_{g}$ is the radius of gyration, $D_{f}$ is the fractal dimension (between 1 and 3), and $k_{f}$ is the fractal pre-factor. Low or high values of the fractal dimension correspond to more rod-like or spherical-like particles, respectively. Several algorithms have been proposed to build morphologies obeying the fractal equation [19,20,21,22]. In this work, we adopt the procedure proposed in References [23,24] based on a particle–cluster aggregation method, which has been verified to generate structures that fulfill the fractal equation also for very few primary particles [24]. We point out that real fractal aggregates can be approximated by coarsened structures where each sphere already represents an agglomerate of smaller real primary particles. In Figure 1, two examples of aggregate morphologies with $N_{p}=20$, $k_{f}=1.3$, and $D_{f}=1.5$ (Figure 1a) or $D_{f}=2.5$ (Figure 1c) are shown. Notice that the algorithm for aggregate generation is based on a sequence of pseudo-random numbers. Hence, by changing the random seed, different morphologies are obtained for the same set of parameters of the fractal equation. Due to the asymmetric shape of the particle, two orthogonal vectors, p and q, are needed to track its orientation. The selection of these orientation vectors will be discussed later. Finally, we denote by Ω and P(t) the fluid and particle domain, respectively. The particle boundary is denoted by ∂P(t) and the surface of the external spherical domain by Σ.

Assuming inertialess conditions and negligible gravity effects, the governing equations for the fluid domain, Ω−P(t), read as follows:

(2) $$\begin{array}{c} \nabla \cdot u=0 \end{array}$$

(3) $$\begin{array}{c} \nabla \cdot \sigma =0 \end{array}$$

(4) $$\begin{array}{c} \sigma =-pI+2\eta (\dot{\gamma })D, \end{array}$$

where u, σ, *p*, I, η, and D are the velocity vector, the stress tensor, the pressure, the 3×3 unity tensor, the viscosity, and the rate-of-deformation tensor $D=(\nabla u+{\left(\nabla u\right)}^{T})/2$, respectively. The viscosity is assumed to be a function of the effective deformation rate defined as $\dot{\gamma }=\sqrt{2D:D}$. Equations (2)–(4) are the mass balance (continuity), the momentum balance and the expression for the total stress, respectively.

In this work, we consider a power-law constitutive equation for the fluid given by:

(5) $$\eta \left(\dot{\gamma }\right)=m{\dot{\gamma }}^{n-1},$$

with *m* the consistency index and *n* the flow index. This model predicts shear-thinning for n<1. For n=1, a Newtonian fluid with (constant) viscosity *m* is recovered.

Concerning the boundary conditions, we impose shear flow at the external spherical surface of the domain:

(6) $$u=({\dot{\gamma }}_{\mathrm{ext}}y,0,0)\;\mathrm{on}\;\Sigma ,$$

with ${\dot{\gamma }}_{\mathrm{ext}}$ the imposed shear flow. No-slip boundary conditions are set on the particle surface resulting in the rigid-body motion equation:

(7) $$u=\omega_{p}\times \left(x-x_{c}\right)\;\mathrm{on}\;\partial P\left(t\right),$$

where x is a point of the surface ∂P(t) and $x_{c}$ is the particle center of volume. As remarked above, the particle translational velocity is irrelevant for the problem under investigation.

To close the set of equations, the hydrodynamic torque acting on the particle needs to be specified. Under the assumptions of inertialess particle and no ‘external’ torques, such balance equation is given by:

(8) $$T=\int_{\partial P(t)}\left(x-x_{c}\right)\times \left(\sigma \cdot n\right)\;dS=0,$$

where T is the total torque on the particle boundary ∂P(t) and n is the unit vector normal to the particle surface pointing from the fluid to the boundary.

The solution of the governing equations gives the fluid velocity and pressure fields, and the particle angular velocity. The orientational dynamics can be computed by integrating the following equation:

(9) $$\frac{d\theta_{p}}{dt}=\omega_{p},$$

with initial condition $\theta_{p}{|}_{t=0}=\theta_{p,0}$.

The governing equations can be conveniently made dimensionless by choosing appropriate characteristic quantities for time, length, and stress. As characteristic length, we select the effective radius of the particle, defined as the radius of a sphere with equivalent volume *V* of the aggregate, $R_{\mathrm{eff}}={\left(\frac{3V}{4\pi }\right)}^{1/3}$. The inverse of the external shear rate $1/{\dot{\gamma }}_{\mathrm{ext}}$ is chosen as characteristic time (giving $R_{\mathrm{eff}}{\dot{\gamma }}_{\mathrm{ext}}$ as characteristic velocity) and $m{\dot{\gamma }}_{\mathrm{ext}}^{n}$ as characteristic stress.

With these characteristic quantities, the dimensionless governing equations and boundary conditions read as:

(10) $$\begin{array}{c} \nabla^{*}\cdot u^{*}=0 \end{array}$$

(11) $$\begin{array}{c} -\nabla^{*}p^{*}+\nabla^{*}\cdot \left(2{\dot{\gamma }}^{*,n-1}D^{*}\right)=0 \end{array}$$

(12) $$\begin{array}{c} u^{*}=\left(y^{*},0,0\right)\;\mathrm{on}\;\Sigma \end{array}$$

(13) $$\begin{array}{c} u^{*}=\omega_{p}^{*}\times \left(x^{*}-x_{c}^{*}\right)\;\mathrm{on}\;\partial P\left(t\right) \end{array}$$

(14) $$\begin{array}{c} T^{*}=\int_{\partial P(t)}\left(x^{*}-x_{c}^{*}\right)\times \left(\sigma^{*}\cdot n\right)\;dS^{*}=0 \end{array}$$

(15) $$\begin{array}{c} \frac{d\theta_{p}}{dt^{*}}=\omega_{p}^{*}, \end{array}$$

where the starred symbols denote dimensionless quantities. Hence, the only dimensionless parameter appearing in the governing equations is the flow index *n*. Of course, we need also to consider the parameters related to the aggregate morphology appearing in Equation (1), that are the number of particles $N_{p}$, the fractal dimension $D_{f}$, and the fractal pre-factor $k_{f}$. Notice that the radius of the primary particles *a* is related to $R_{\mathrm{eff}}$ through $N_{p}$. Also, the radius of gyration $R_{g}$ is determined once the aforementioned three parameters are specified. In what follows, all the symbols (without stars) will refer to dimensionless quantities.

Aim of this work is to predict the viscosity of a dilute suspension of fractal-like aggregates in a power-law fluid. In the dilute regime, the suspension viscosity can be expressed as:

(16) $$\eta =\eta_{s}\left(1+B\;\phi \right),$$

where $\eta_{s}$ is the matrix viscosity defined by Equation (5), ϕ is the (low) particle volume fraction, and *B* is the intrinsic viscosity. Once the local stress field is calculated by solving the governing equations, the intrinsic viscosity is computed through the numerical homogenization procedure as described in References [16,25]. In brief, this procedure consists in evaluating first the average power density:

(17) $$\langle P\rangle =\langle \sigma :D\rangle =\frac{1}{V_{0}}\int_{V_{f}}\sigma :D\;dV,$$

where $V_{0}$ and $V_{f}$ are the suspension and fluid volumes. The suspension viscosity is, then, evaluated as:

(18) $$\eta =\frac{\langle P\rangle }{2D_{\mathrm{ext}}:D_{\mathrm{ext}}}=\frac{\langle P\rangle }{{\dot{\gamma }}_{\mathrm{ext}}^{2}},$$

with $D_{\mathrm{ext}}$ the rate-of-deformation at the external boundaries of the domain that, for a simple shear flow, reduces to the last term of Equation (18). From the suspension viscosity, we can readily evaluate the intrinsic viscosity *B* as:

(19) $$B=\frac{\eta -\eta_{s}}{\eta_{s}\;\phi }.$$

Due to the anisotropic particle shape, the intrinsic viscosity depends on the particle orientation. As we will show later, the orientational dynamics is rather complex and neither a steady-state nor a simple periodic regime is achieved. Hence, we integrate the instantaneous intrinsic viscosity over the particle orbit for a sufficiently long time *T* so that the average value, defined as:

(20) $$\bar{B}=\frac{1}{T}\int_{0}^{T}B\left(t\right)dt,$$

does not change within a tolerance (chosen as 1%). For each particle morphology, we run several simulations for different initial orientations. The time-average intrinsic viscosity is, then, averaged over *M* initial orientations to get:

(21) $$\langle \bar{B}\rangle =\frac{1}{M}\sum_{M}\;\bar{B}.$$

Finally, to make the results independent of the seed used to generate the aggregates, we repeat the simulations for different seeds for fixed values of the fractal parameters, and define the ensemble-average intrinsic viscosity as:

(22) $${\langle \bar{B}\rangle }_{m}=\frac{1}{N_{\mathrm{seed}}}\sum_{N_{\mathrm{seed}}}\;\langle \bar{B}\rangle ,$$

with $N_{\mathrm{seed}}$ the number of seeds.

In this work, we fix the fractal pre-factor $k_{f}=1.3$, which is a value commonly used in the literature to describe realistic aggregate morphologies [26]. The suspension intrinsic viscosity is, then, studied by varying the power-law index, the number of primary particles forming the aggregate, and the fractal dimension.

### 2.2. Numerical Method

Except for the simplest case of spherical particles, the intrinsic viscosity is a function of the particle orientation. Hence, the calculation of $\bar{B}$ in Equation (20) requires the knowledge of the evolution of the particle orientation dynamics. To this aim, starting from an initial orientation, the governing Equations (10)–(14) should be solved at each time step followed by the integration of the kinematic Equation (15). This procedure is time-consuming since, as we will see later, the final integration time must be 3–4 orders of magnitude higher than the characteristic time in order to get an accurate estimate of the intrinsic viscosity. Furthermore, for each aggregate morphology, the procedure should be repeated for several initial orientations. However, since no time-derivatives appear in Equations (10)–(14), a strategy to speed-up the simulations has been recently proposed [27]. We run single-step simulations for several orientations of the particles (i.e., without performing any time-integration). From these simulations, we build a look-up table containing the particle angular velocity and the intrinsic viscosity for each orientation. Then, the orientation dynamics is reconstructed by solving the kinematic Equation (15) where the angular velocity on the right-hand side is taken by interpolating the simulation data. Once the look-up table has been constructed, it can be used for calculating the particle orbits for any initial orientation, thus greatly reducing the computational cost.

Let us describe in more detail the adopted procedure. Since the particle shape has no symmetry axes, two (orthogonal) orientation vectors p and q are required to identify the orientation of the particle in a fixed frame. The choice of these two vectors to build the look-up table is done as follows. Let us consider the unit vector $\hat{x}=\left(1,0,0\right)$ in the reference frame of the aggregate generated by the particle-cluster method [23,24]. Notice that this vector identifies an arbitrary direction (depending on the algorithm used to build the aggregate) and, as such, does not have any physical meaning. A triangulated icosahedral mesh over the unit sphere is generated, that is, the unit sphere is divided in triangles with icosahedral symmetry. The aggregate is then rigidly rotated so that the orientation vector p, originally aligned with the unit vector $\hat{x}$, is brought to coincide with the vertices of the triangulated mesh.

For each of these orientations p, we select the vectors q as the equally-distributed vectors over the unit circle around p. Specifically, the calculation of the vectors q is carried out by first defining $N_{q}$ vectors $q_{xy,i}$ uniformly distributed over the unit circle in the xy− plane, that is, $q_{xy,i}=\left(\cos\psi ,\sin\psi ,0\right)$ with $\psi =-\pi +2\pi \left(i-1\right)/N_{q}$ for $i=1,...,N_{q}$; then, the vectors q are computed as $q_{i}=R_{\hat{z},p}\cdot q_{xy,i}$ where $R_{\hat{z},p}$ is the rotation matrix transforming $\hat{z}=\left(0,0,1\right)$ to p. In this way, the vectors q are orthogonal to p (since $q_{xy}$ are orthogonal to $\hat{z}$) and the vector $q_{1}$ corresponds to the transformation of $-\hat{x}$ according to the rotation matrix $R_{\hat{z},p}$. In summary, the aggregate generated by the particle-cluster method is first rotated so that p coincides with one vertex of the icosahedral mesh, and then it is further rotated around p to get one of the $N_{q}$ vectors q as discussed above.

We select an icosahedral subdivision with triangulation number $T_{n}=4$ resulting in 42 vertices over the unit sphere and $N_{q}=12$ for a total of 504 entries (i.e., single-step simulations) in the look-up table. In this way, the angular distance of p on the unit sphere and of q on the unit circle (around p) is approximately the same (about π/6). We have verified that this subdivision is sufficient to assure a good accuracy of the interpolation.

Once the look-up table has been computed, integration of Equation (15) is done by interpolating the angular velocity. Notice that two kinds of interpolations need to be performed, that is, one over the unit sphere (for p) and one over a unit circle orthogonal to p (for q). After obtaining the angular velocity, we update both orientation vectors instead of the rotation angle $\theta_{p}$ by using quaternions [28]. The entire procedure (interpolation and orientation update) is summarized in Algorithm 1.
**Algorithm 1** Procedure used to update the particle orientation dynamics1:$p_{0},q_{0}\leftarrow$ initial orientation2:**for**t←1,num_time_steps**do**3:    Identify the spherical triangle containing p with vertices $v_{i}\;\left(i=1,2,3\right)$4:    **for** i←1,3 **do**5:        Compute the rotation matrix $R_{p,v_{i}}$ from p to $v_{i}$6:        $q_{i}^{\prime }\leftarrow R_{p,v_{i}}\cdot q$7:        Compute $\omega_{i}$ and $B_{i}$ by interpolating the look-up table with entries $v_{i}$ and $q_{i}^{\prime }$8:    **end for**9:    Compute ω and *B* by interpolating $\omega_{i}$ and $B_{i}$ over the spherical triangle in p10:    Update p and q using quaternions.11:**end for**

The same triangular mesh chosen for building the look-up table is used for interpolation. First of all, the triangle containing p needs to be identified (step 3 of Algorithm 1). We use the search procedure described in Reference [29]. The interpolation of the angular velocity and intrinsic viscosity inside a spherical triangle mesh requires the knowledge of these quantities at the triangle vertices. To do this, we compute the rotation matrix needed to rotate p on the unit vectors corresponding to the triangle vertices $v_{i}$ and apply this rotation matrix to q in order to get the values $q_{i}^{\prime }$ in these vertices (steps 5 and 6). Then, we enter into the look-up table with $v_{i}$ and $q_{i}^{\prime }$ to perform the interpolation (step 7). Notice that the values $v_{i}$ are already present in the look-up table since, as mentioned above, the same mesh is used for building the table and for performing the interpolation. This is not the case for $q_{i}^{\prime }$. Hence, a mono-dimensional interpolation is required over the block of $N_{q}$ data in the table. This is done by using a quadratic interpolation. At the end of the inner loop in Algorithm 1 we get the angular velocity and the intrinsic viscosity in the three vertices of the triangle. A linear interpolation inside the spherical triangle over p is finally performed to obtain the values of the two quantities in the desired point [29,30] (step 9). Once the angular velocities are known, the quaternions are updated in time by using a third-order Adams-Bashforth scheme. The rotation matrix in terms of quaternions is constructed and used to update p and q (step 10).

The one-step direct numerical simulations are carried out by solving the governing equations by the finite element method. The particle angular velocity are treated as additional unknowns, and are included in the weak form of momentum equation. The torque-free condition is imposed through Lagrange multipliers in each node of the particle surface [31].

The spherical primary particles forming the aggregate generated by the particle-cluster method are tangent. To avoid numerical problems in the region of the contact point, we perform a Boolean union operation of the spheres with a set of cylinders connecting the centers of the spheres in contact (that are readily identified since the generation algorithm provides the connectivity list). The radius of the cylinders is chosen 0.732a. We have verified that, by reducing the cylinder radius, the rotational dynamics and the resulting intrinsic viscosity is weakly affected. The aggregate surface is, then, smoothed and meshed. These operations are performed through the library PyMesh [32]. Examples of the surface meshes for the aggregates in Figure 1a,c are shown in Figure 1b,d. The volume mesh, that is the mesh between the external spherical domain and the aggregate, is made by tetrahedral elements and is generated by Gmsh [33].

Mesh and time convergence, the dimension of the external spherical domain as well as the validity of the procedure described above are carefully checked. The parameters used in the simulations are reported in Table 1 where Δx and $\Delta R_{\mathrm{ext}}$ (made dimensionless by the primary particle radius *a*) are the size of the elements on the aggregate surface and the external domain, respectively, $R_{\mathrm{ext}}$ is the radius of the external sphere, and $N_{\mathrm{elem}}$ is the number of tetrahedral elements in the volume mesh. We verified that, by reducing Δx and $\Delta R_{\mathrm{ext}}$ and increasing $R_{\mathrm{ext}}$ by about 20%, the particle angular velocity and the intrinsic viscosity change by less than 1.5%. The time-step size is fixed at Δt=0.05, giving the same orientation dynamics by repeating the computation with Δt=0.025. We have also verified the accuracy of the solution of the governing equations for a spherical particle in a power-law fluid and the validity of the homogenization procedure for the most critical case of n=0.4 by comparing the results with those reported in Reference [25] (deviations are less than 2%). Finally, the procedure described in Algorithm 1 is applied to a spheroid. The particle dynamics and the time-dependent intrinsic viscosity is in excellent agreement with the Jeffery results [7].

## 3. Results

In this section we present results by varying the fractal dimension, the flow index, and the number of primary particles. Specifically, we consider three values for each parameter, that is, $D_{f}=\left[1.5,2.0,2.5\right]$, n=[1.0,0.7,0.4], and $N_{p}=\left[10,20,50\right]$. Notice that, since the intrinsic viscosity is normalized with respect to the aggregate volume, the number of primary particles defines the structure resolution (finer for many particles) and, for low fractal dimension, it is also connected to the aggregate aspect ratio.

**Figure 2:**
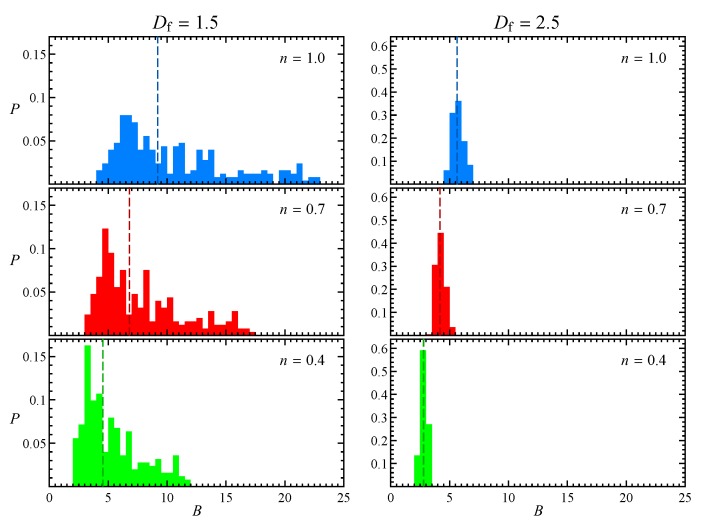
Probability distribution of the intrinsic viscosity corresponding to the initial aggregate orientations used to build the look-up table, for low (**left**) and high (**right**) fractal dimension and three flow indexes. The dashed line represents the median of the distribution.

Figure 2 shows the probability distribution of the intrinsic viscosity with respect to the same initial orientations considered to build the look-up table, for the two aggregates reported in Figure 1 and three flow indexes. The dashed lines represent the medians of the distributions, which are all approximately unimodal. Comparing the results for different fractal dimensions, it can be seen that at lower $D_{f}$ the distribution of the intrinsic viscosity is wider, as the particles are more anisotropic and their orientation becomes more relevant. In particular, *B* is higher when the aggregate is aligned with the gradient direction (i.e., *y*), and lower when it is aligned with the flow or vorticity direction, similarly to prolate spheroids. On the other hand, the distribution variance reduces at higher $D_{f}$ as the particles are more sphere-like. In agreement with the results for a sphere in power-law fluid [25], shear-thinning determines a reduction of the intrinsic viscosity, but the shape of the distribution remains the same.

**Figure 3:**
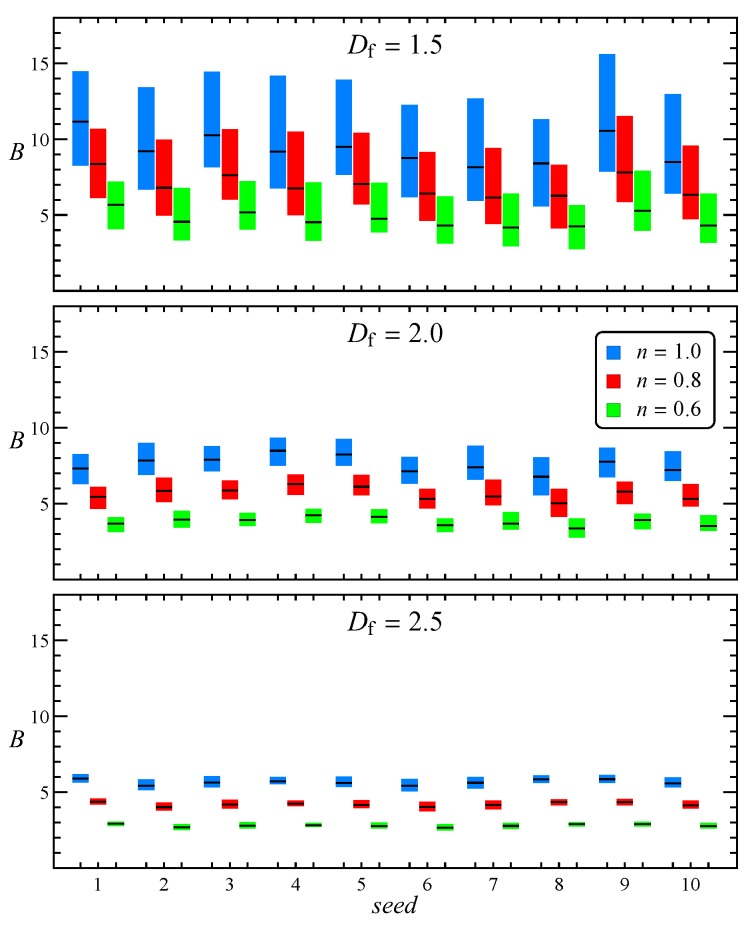
Box plot of the intrinsic viscosity as a function of aggregate random seed, flow index and fractal dimension. The black dash within each box represents the median of the distribution.

As stated above, to make the results independent of the specific random seed used to generate the aggregates, for every combination of the other parameters, we repeat the simulations with ten different seeds. The effect of the specific morphology on the intrinsic viscosity distribution is reported in Figure 3 with a box plot showing the first, second (i.e., the median), and third quartile of every distribution. In all cases we find again the same trend seen in Figure 2 with fractal dimension and flow index, whereas the effect of the random seed is of secondary importance.

**Figure 4:**
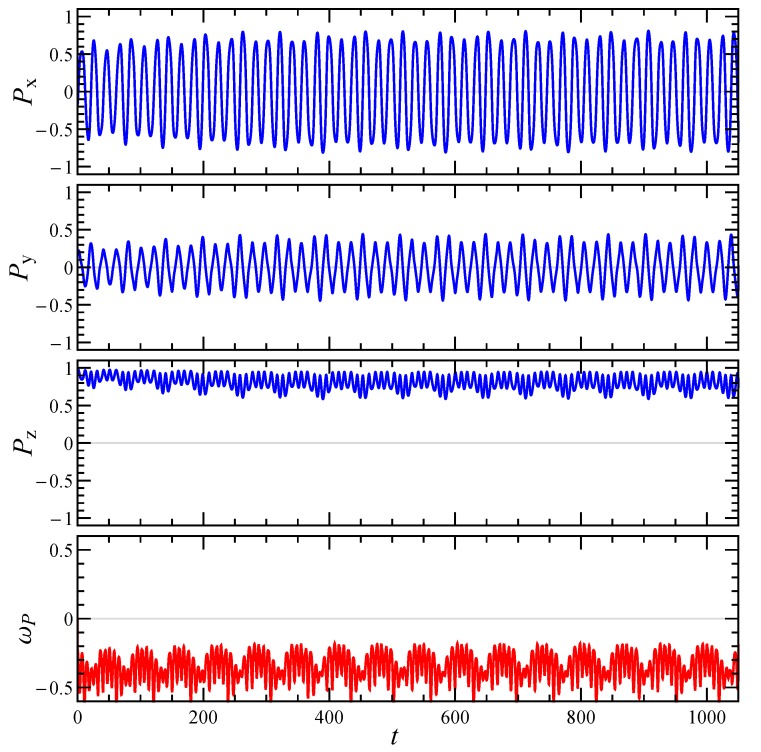
Time evolution of the components of the particle smallest principal axis of inertia P and of the angular velocity around it $\omega_{P}$, for an initial orientation close to the vorticity axis *z*. The aggregate is the one reported in Figure 1b, the other parameters are $N_{p}=20$, $D_{f}=1.5$, and n=1.0.

**Figure 5:**
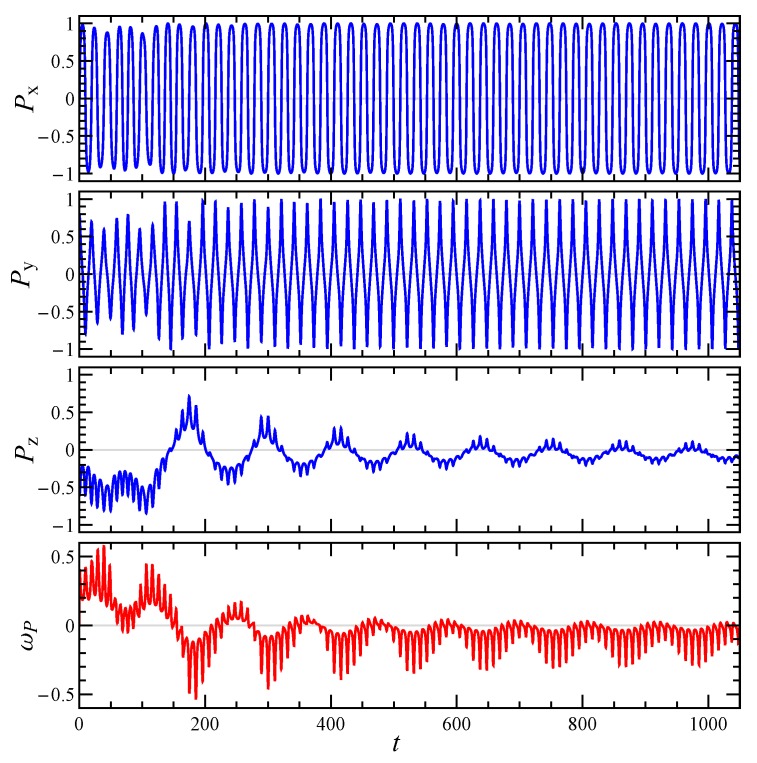
Time evolution of the components of the particle smallest principal axis of inertia P and of the angular velocity around it $\omega_{P}$, for an initial orientation far from the vorticity axis *z*. The aggregate is the one reported in Figure 1b, the other parameters are $N_{p}=20$, $D_{f}=1.5$, and n=1.0.

Since the intrinsic viscosity depends on the particle orientation, which changes with time due to the imposed shear flow, it is important to reconstruct the particle dynamics. The rotational dynamics of an irregularly shaped aggregate can be better understood by looking at its principal axes of inertia. Specifically, let’s denote with P the principal axis corresponding to the smallest moment of inertia, which for an elongated particle (small $D_{f}$) corresponds to its ‘natural’ orientation. Figure 4 and Figure 5 show the time evolution of the Cartesian components of P and the angular velocity around it $\omega_{P}=\omega_{p}\cdot P$, for the aggregate reported in Figure 1b and two initial orientations. In both cases the orientational dynamics is rather complex and neither a steady-state nor a simple periodic regime is achieved. Two periods can be recognized, one shorter connected to the rotation around the vorticity axis *z*, and one longer connected to the rotation around P. When the particle starts with an orientation close to the *z*-axis (see Figure 4), it undertakes a kayaking-like dynamics with approximately elliptical orbits around the *z*-axis, more elongated in the *x*-direction. On the other hand, when the initial orientation is sufficiently far from the *z*-axis (see Figure 5), the particle tends to align with the shear plane, that is, $P_{z}$ progressively reduces, similar to a tumbling motion. At the same time also $\omega_{P}$ decreases, thus determining an extension of the rotation period around P. The same qualitative behavior of the aggregate dynamics is observed for shear-thinning fluids.

**Figure 6:**
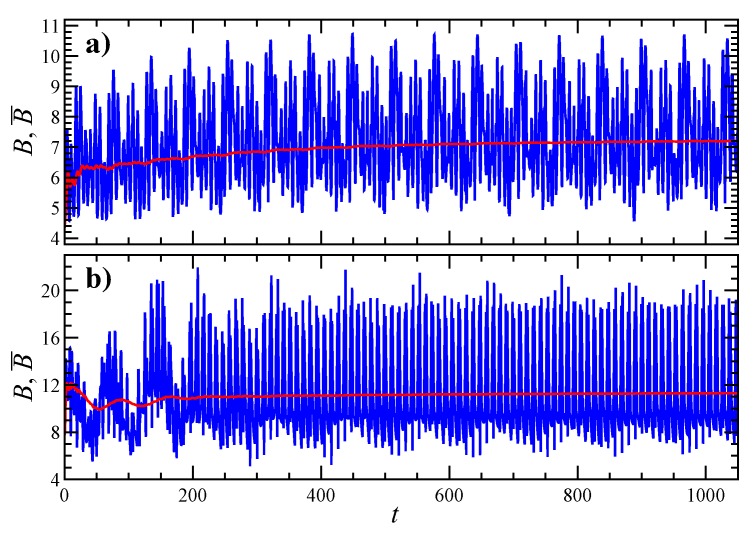
(**a**) Time evolution of intrinsic viscosity *B* (blue curve) and time-average intrinsic viscosity $\bar{B}$ (red curve) for the trajectory reported in Figure 4. (**b**) The same as **a** for the trajectory in Figure 5.

Once the particle orientational dynamics is known, the time evolution of intrinsic viscosity *B* and time-average intrinsic viscosity $\bar{B}$ can be reconstructed. Figure 6 shows the trend of *B* and $\bar{B}$ for the trajectories seen in previous figures. Specifically, panel Figure 6a refers to Figure 4 (where the particle undertakes kayaking) and panel Figure 6b refers to Figure 5 (where the particle tumbles close to the shear plane). In both cases, while *B* continues to oscillate due to the aforementioned dynamics, after a certain time, $\bar{B}$ reaches a steady state condition. Notice that such steady state values are different for the two initial orientations considered. $\bar{B}$ is lower in Figure 6a since the aggregate is mainly aligned with its longest axis towards the vorticity direction. Conversely, $\bar{B}$ is higher in Figure 6b since, during the aggregate tumbling motion, P periodically passes close to the gradient direction, which corresponds to the maximum intrinsic viscosity.

**Figure 7:**
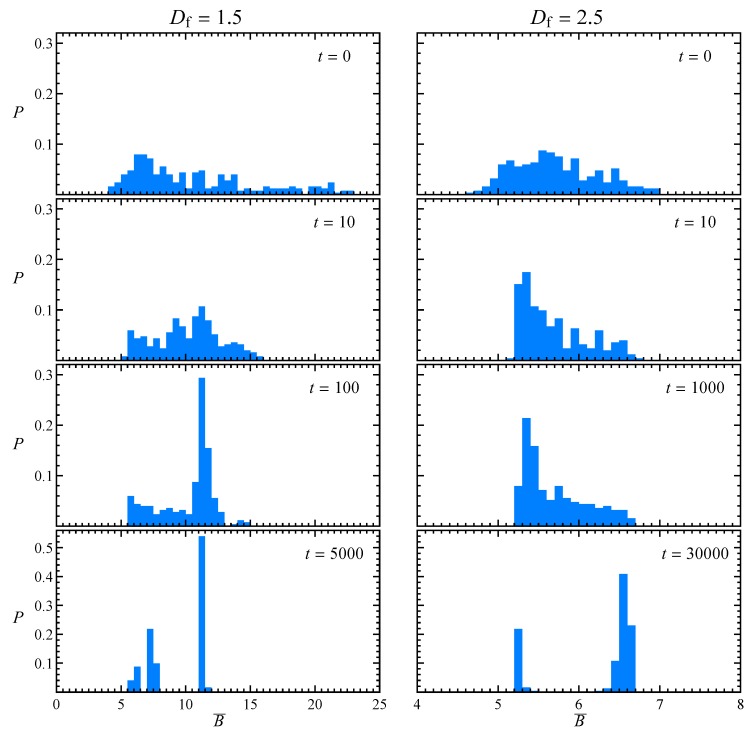
Probability distribution of the time-average intrinsic viscosity $\bar{B}$ corresponding to the initial aggregate orientations used to build the look-up table and n=1.0, for low (**left**) and high (**right**) fractal dimension, parametric in time.

By repeating this procedure for many orientations, it is possible to obtain the evolution of the probability distribution of the time-average intrinsic viscosity, as reported in Figure 7 for the usual two aggregates and n=1.0. Notice that the initial distributions are the same of Figure 2. As just seen in the previous figure, for low fractal dimension more than one steady state value is present, with the majority of initial orientations leading to dynamics like the one in Figure 6b with $\bar{B}\approx 11.5$. The two lower peaks visible for $D_{f}=1.5$ at t=5000 correspond to P approximately aligned with the positive (shown in Figure 4) or negative (not shown) vorticity direction. These last two peaks are close but not equal since the aggregate has no symmetries. A similar behavior can be observed at high fractal dimension, but with a longer time needed to reach the steady state. The two peaks visible for $D_{f}=2.5$ at t=30,000 are still related to the kayaking and tumbling motion. However, those related to alignment of P to the vorticity direction or its opposite are not distinguishable because of the more isotropic particle shape.

**Figure 8:**
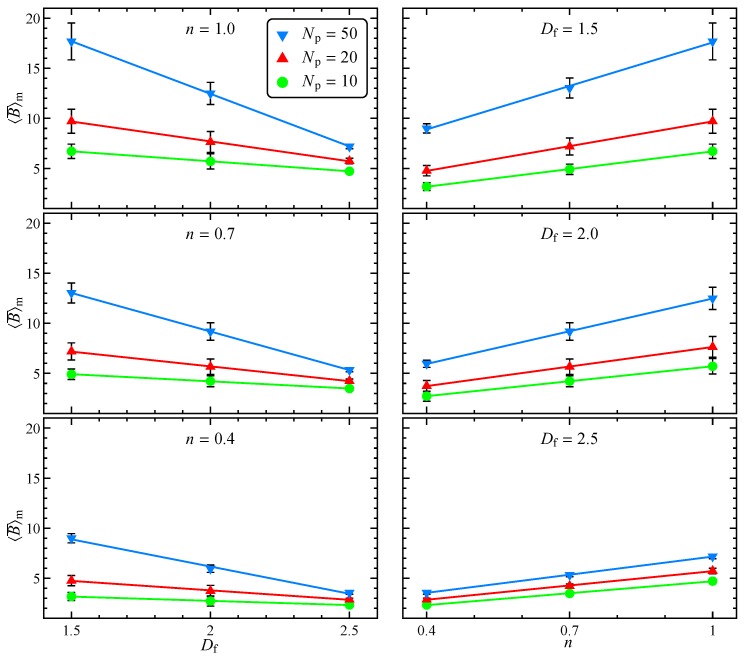
Ensemble-average intrinsic viscosity as a function of fractal dimension, parametric in the number of aggregate primary particles and for different flow indexes (left). Same data as a function of flow index for different fractal dimensions (right). The data standard deviation and trend line are also reported.

Finally, averaging on all the initial orientations and random seeds we get the ensemble-average intrinsic viscosity defined in Equation (22). Figure 8 reports the dependency of ${\langle \bar{B}\rangle }_{m}$ on the fractal dimension and flow index, parametric in the number of primary particles, together with standard deviation and trend line. In the range considered, ${\langle \bar{B}\rangle }_{m}$ decreases with $D_{f}$ and increases with $N_{p}$. The decreasing trend of ${\langle \bar{B}\rangle }_{m}$ with the fractal dimension, previously observed for Newtonian fluids [11], is related to the aggregate shape and can be justified recalling that the intrinsic viscosity of a suspension of rod-like particles is higher than the one for a suspension of spheres [7]. For the same reason, the number of primary particles forming the aggregate weakly affects the intrinsic viscosity at high fractal dimension. On the contrary, $N_{p}$ has a strong influence on ${\langle \bar{B}\rangle }_{m}$ for low values of $D_{f}$. Indeed, for a sphere-like aggregate the number of primary particles only defines its resolution, whereas for a rod-like aggregate it is connected to the aspect ratio that, in turn, determines the viscosity of the suspension [7]. As regarding the effect of the flow index, in agreement with the previous literature for suspensions of spherical and spheroidal particles [14,16,25], shear-thinning reduces the intrinsic viscosity. Specifically, as visible on the right column of Figure 8, in the investigated range the intrinsic viscosity linearly depends on the flow index. As expected standard deviation is lower for high fractal dimensions, since both initial orientation (see Figure 2) and random seed (see Figure 3) have a relatively minor effect.

The intrinsic viscosity shown in the previous figures accounts for the effective volume of the aggregate that, as discussed in Section 2.1, is computed from the union of spheres and cylinders. An alternative approach is to normalize the intrinsic viscosity by the aggregate hydrodynamic volume that, for a set of spherical particles with radius *a*, is the volume of a sphere with radius $R_{H}\;a$, where $R_{H}$ is the hydrodynamic radius [11]. The hydrodynamic radius is defined as the radius of a sphere that gives the same drag force acting on the aggregate in a uniform flow [34], and, in a Newtonian fluid, it is related to the eigenvalues of the translational mobility tensor. Neglecting the contribution of the connecting cylinders, the ensemble-average intrinsic viscosity normalized with the hydrodynamic volume is then:

(23) $${\langle \bar{B}\rangle }_{m,H}=\frac{N_{p}}{R_{H}^{3}}{\langle \bar{B}\rangle }_{m}.$$

**Figure 9:**
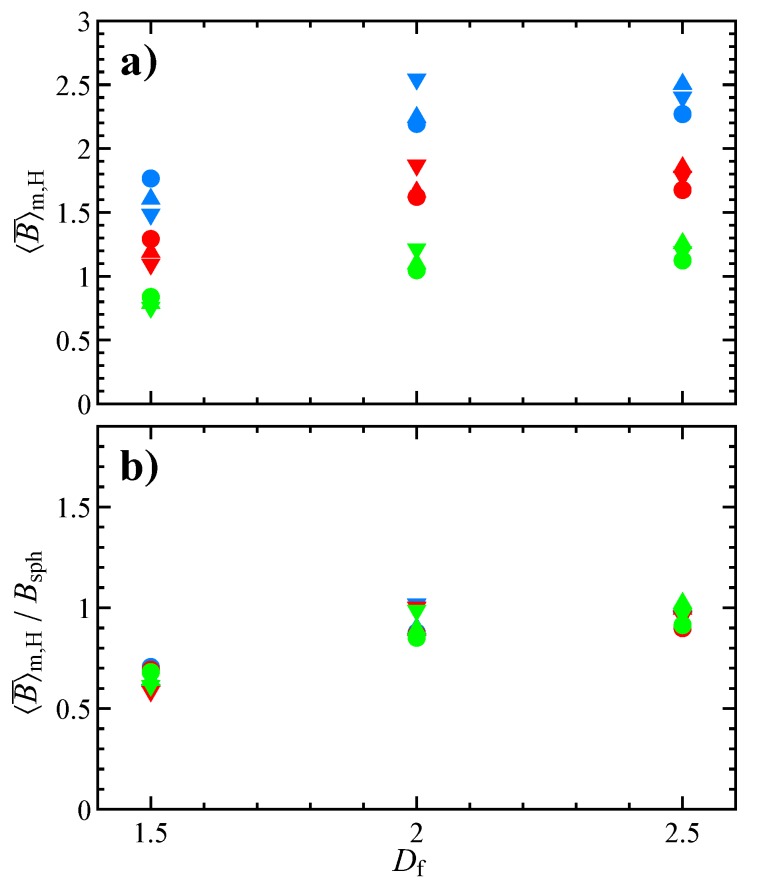
(**a**) Ensemble-average intrinsic viscosity normalized with the aggregate hydrodynamic radius as in Equation (23). (**b**) Same data as panel **a** normalized by the intrinsic viscosity of a sphere in a power-law fluid. In both panels, the symbols denote the number of primary particles composing the aggregate (lower triangle $N_{p}=50$, upper triangle $N_{p}=20$, and circle $N_{p}=10$) and the colors refer to the flow index (blue n=1.0, red n=0.7, and green n=0.4).

Figure 9a shows the trend of ${\langle \bar{B}\rangle }_{m,H}$, in which the values of $R_{H}$ are taken from the literature [23,34]. Some remarks are in order: (i) Equation (23) and the used hydrodynamic radii assume that the aggregate is made of tangential spherical particles, (ii) the values of $R_{H}$ are calculated for an aggregate suspended in a Newtonian fluid (notice that the calculation of the hydrodynamic radius for a power-law fluid is not straightforward as, due to the non-linearity of the constitutive equation, the mobility tensor cannot be used for its evaluation). Despite these approximations, the data scale fairly well with respect to the number of primary particles (symbols with the same color in figure). As a consequence of the previous normalization, at high fractal dimension ${\langle \bar{B}\rangle }_{m,H}$ tends to the value for a spherical particle (i.e., 2.5 in the Newtonian case) [11]. This motivates us to further normalize the data with respect to the intrinsic viscosity of a dilute suspension of spheres in a power-law liquid, given by $B_{\mathrm{sph}}=0.383+2.117n$ [14,25,35]. As visible in Figure 9b, all the data collapse on a single curve. Hence the viscosity of a dilute suspension of aggregates (with the same fractal parameters) in a power-law fluid is completely determined by the fractal dimension. Of course, the prediction of the intrinsic viscosity form the master trend in Figure 9b requires the knowledge of the hydrodynamic radius of the aggregate population.

## 4. Conclusions

The viscosity of a dilute suspension of fractal aggregates in a shear-thinning fluid has been investigated. The aggregate morphology is generated through a particle-cluster method combining spherical particles with equal size in order to satisfy the fractal equation. The power-law constitutive equation is used to model the fluid. Finite element simulations are employed to solve the fluid dynamics problem of an aggregate in an unbounded shear flow for a fixed particle orientation. The simulation gives the velocity and pressure fields, and the angular velocity of the aggregate. A homogenization procedure is adopted to obtain the intrinsic viscosity. The simulations are run for a uniform distribution of all the possible orientations, building a database of angular velocities and intrinsic viscosities. The orbits followed by the aggregate are, then, reconstructed by integrating the particle kinematic equation with angular velocity interpolated from the database. Finally, the intrinsic viscosity computed along the orbit is averaged in time and over several initial orientations and seeds used to build the morphology.

The rotational dynamics of the aggregate is rather complex, characterized by irregular oscillations and more than one characteristic period. At long times, the aggregate approximately aligns with one of its principal axes of inertia to the vorticity direction, performing a kayaking motion. Hence, multiple regimes depending on the initial particle orientation are possible, thus leading to different time-average intrinsic viscosities. The ensemble-average intrinsic viscosity decreases by increasing the fractal dimension, that is, from rod-like to sphere-like aggregates. For low values of the fractal dimension, the number of particles forming the aggregate directly affects the aspect ratio and, in turn, leads to relevant variations of the intrinsic viscosity. Shear-thinning reduces the intrinsic viscosity showing a linear dependence with the flow index in the investigated range. Finally, the intrinsic viscosity data, normalized through the hydrodynamic volume and the intrinsic viscosity of a dilute suspension of spheres in a power-law liquid, collapse on a single curve in terms of the fractal dimension.

The results presented in this work help us to understand the effect of both complex particle shape and non-Newtonian rheology on the intrinsic viscosity of suspensions in the absence of interparticle hydrodynamic interactions. Hence, in some sense, they represent the extension of Einstein result to a (dilute) suspension of fractal aggregates in a power-law fluid. Furthermore, they can be used as starting point to estimate the suspension bulk viscosity at high volume fractions [25,35,36].

## Figures and Tables

**Table 1:** Mesh and geometrical parameters used in the simulations.

$N_{p}$	Δx	$R_{\mathrm{ext}}$	$\Delta x_{\mathrm{ext}}$	$N_{\mathrm{elem}}$
10	0.20	40	10	∼20,000
20	0.25	40	10	∼20,000
50	0.30	50	20	∼30,000
